# Transplantology: Challenges for Today

**DOI:** 10.1007/s00005-016-0439-1

**Published:** 2017-01-12

**Authors:** Maria Boratyńska, Dariusz Patrzałek

**Affiliations:** 1 Department of Nephrology and Transplantation Medicine, Wroclaw Medical University, Wrocław, Poland; 2 Department of Clinical Basics of Physiotherapy, Faculty of Health Science, Wroclaw Medical University, Wrocław, Poland

**Keywords:** Clinical transplantation, Organ donation, Chronic rejection, Tolerance

## Abstract

Clinical transplantology in Poland had its 50th anniversary this year. With the early and long results comparable to the best achieved in the world leading centers, we face old and completely new challenges for this medical speciality. Main and growing challenge is insufficient number of available organs. With less than 15 donors/mln population/year Poland stay in the lower row of European countries in this measurement of transplant activity. Donation system is not efficient enough and we lose a big number of potential donors still. Living donation (with the exception for the fragments of the liver) remains low despite of different initiatives made so far on the national and local levels. Donation after cardiac death is possible from the point of Polish juridical regulations, but since last 3 years had not showed real impact on country donation rates (only three procedures done). Methods of tissue typing remain slow and cause relatively long times of cold ischemia for kidney programs. Second main challenge is chronic rejection causing loss of organs in the long-term follow-up and no efficient treatment employed. The emerging possibility of tolerance induction despite of plenty of new protocols proposition in the publications does not show up a clinical everyday practice in work. The same is with xenotransplantation promises; even we were informed recently that till 2030 such genetically modified porcine organs will be available. The next challenge is production of organs and tissues from own recipients cells installed on the different scaffolds or 3D printed. Other challenge is the personnel working in this field. We observe like in the other European countries lack of new candidates for work in this field together with serious problems of nursing staff, being a catastrophic perspective in country medical service in general, not only in transplant centers. The last but not least challenge is financial side of transplant programs.

## Introduction

Clinical transplantology in Poland had its 50th anniversary this year. With the early and long results comparable to the best achieved in the world leading centers, we face old and completely new challenges for this medical speciality. In this paper, we wanted to present the most important problems of clinical transplantology as they are seen from Polish perspective. Our aim was to show the new area for scientific research for people involved in basic sciences in immunology and immunogenetics during EWIC conference held in Wroclaw in April 2016.

The main challenges in today’s transplantology are insufficient number of available organs, chronic rejection, clinical implementation of tolerance induction, practical application of xenotransplantation, regenerative medicine using organs produced with own recipient cells, human resources in transplantation staff, and finances involved in transplantation programs on the country level.

## Organ Donation

Despite many efforts Poland rate in donation measured traditional as number of donors per million population per year is not exceeding 15 (Malanowski and Czerwiński 2016). The recent tendency is showing further decrease. The reasons for such situation are multifactorial. The first source of organs is brain dead donors. The number of such patients is decreasing everywhere in the world (less cranial trauma, better medical care for cerebrovascular accident), but still the main problem in Poland is small percent of potential donors identification and less than 40% of hospitals show any donation activity. The weak position of transplant coordinator in most of Polish hospitals is added to the above mention problems.

What is more the number of donors family refusals is growing from 10 to over 20%, mainly because of negative presentations in media—negating the brain death and diagnostic protocols.

The second source of organs is donors after cardiac death (DCD). It is allowed by law in Poland but the whole procedure is “transplant effective” in less than 40% and consumes a huge number of trained staff, additional equipment and materials. Polish governmental refundation system is not ready for such high expenses yet. As a result, there were only three DCD procedures done in Poland during last 3 years, when the law for such procedure was introduced. The third source of organs (only kidneys and liver fragments) comes from living related donors. Kidneys from living donation in Poland represent less than 2% of kidney transplantations. This small number comes from high rate of potentials living donors discard ratio and social reluctance for living donation—both from recipients, their families and medical staff in dialysis centers. It seems that for all above mentioned problems we need a complex and continuous work to better adapt whole system to better serve the still growing needs.

## Chronic Allograft Rejection

Chronic allograft rejection (referred as injury or dysfunction) is a multifactorial process, which leads to allograft fibrosis and failure. After death of a patient with a functional graft, it is the second most common cause of graft loss (except liver transplantation). Renal allograft failure is a main cause of return of patients to dialysis treatment and requirement for another transplant. On the poltransplant waiting list, 30% of patients await a second or third kidney transplant. Currently, the immunosuppressive regimens unselectively inhibit the activity of T and B cells, by interfering with their effector and immunoregulatory functions and they do not fully control the chronic rejection reaction.

Chronic allograft rejection may develop months or years after transplantation, but the incidence increases with time after transplantation. Our study found that chronic renal allograft dysfunction occurred in 43% of recipients within 10 years after transplantation (Boratynska et al. 2014). The studies from other transplant centers showed chronic allograft nephropathy in 55–62% of patients after about 8 years from renal transplantation (Grinyo et al. 2011; Marcén et al. 2010). High incidence of chronic rejection defined as cardiac allograft vasculopathy occurs in 25–60% of heart recipients and as obliterative bronchiolitis in 28–80% of lung transplant patients within 5 years after transplantation. However, chronic rejection affects only about 4–6% of liver transplant patients.

The clinical diagnosis is suggested by gradual deterioration of graft function and depends on transplanted organ. Cardiac vasculopathy characterized by occlusive narrowing of coronary vessels is manifested as coronary heart disease. Chronic renal allograft injury is characterized by a gradual decline in glomerular filtration rate, proteinuria and arterial hypertension (Josephson 2014; Nankivell and Kuypers 2011).

Histological changes usually precede clinical symptoms of chronic renal allograft injury. Wavamunno et al. (2007) found endothelial and subendothelial ultrastructural abnormalities in glomerular and peritubular capillaries present as early as 1 month after transplantation, and light microscopy changes at 2.3 years in patients that many years later developed transplant glomerulopathy. Nankivell et al. (2003) revealed that two-thirds of renal allograft fibrosis observed at 10 years were already present in the first year after transplantation. The common pathological changes observed in chronic renal allograft injury are interstitial fibrosis, tubular atrophy with accompanying vascular abnormalities (endothelial inflammation and injury which leads to thickening of vessel walls by accumulating connective tissue, mononuclear infiltration, proliferation of myofibroblasts), and glomerulopathy (thickening of glomerular capillary walls, segmental or global sclerosis). The chronic renal allograft injury is the final pathway of nephron injury with its fibrotic healing response (Chapman et al. 2005).

The major mechanisms of chronic rejection involve delayed type hypersensitivity, innate immunity, chronic antibody-mediated rejection, and immunoregulatory response within the graft. Wedel et al. (2015) presented new look on the molecular events within the intragraft microenvironment that defines the phenotype of rejection and sustains chronic rejection process. They propose that damage of endothelial cells resulting from ischemia reperfusion injury, T cell cytotoxicity, and alloantibodies. These elicit cascade of events leading to local expression of cytokines and several proangiogenic growth factors (e.g., vascular endothelial growth factor—VEGF, stromal cell-derived factor 1) and mononuclear cell infiltration that promote local tissue hypoxia through the transcriptional activity hypoxia-inducible factor 1 α (HIF-1α). All these events precede the process of endothelial to mesenchymal transition that is associated with collagen production and fibrosis development. VEGF plays a central pathophysiological role within the allograft; it mediates vascular remodeling and interacts with VEGF receptors on lymphocyte subsets. It facilitates transmigration activated T cells and potentiates inflammation. In models of acute rejection, antibodies to VEGF prolong graft survival. The investigators indicated that new molecules regulated by HIF-1α dependent path, semaphorins, and neurophilins, as well as mTOR/Akt signaling can drive the chronic rejection process (Wedel et al. 2015).

In cardiac and renal allografts, chronic antibody-mediated rejection (AMR) plays a significant role in allograft injury and transplant loss (Colvin 2007; Costello et al. 2013; Smith and Colvin 2012). AMR is characterized by development of donor-specific alloantibodies and histological damages. These in kidney transplant include thrombotic microangiopathy, macrophage and granulocyte margination in peritubular capillaries and within the glomeruli, and basement membrane multilamination (Colvin 2007). We and other investigators observed de novo anti-HLA antibodies in 30–50% of patients with AMR, subsequently half of them lost transplant (Banasik et al. 2013; Einecke et al. 2009; Hidalgo et al. 2009). Our data also revealed high prevalence of non-HLA antibodies, such as anti-endothelial cell antibodies, anti-angiotensin II type 1 receptor antibodies (anti-AT1R), and anti-endothelin receptor antibodies (anti-ETAR) at fifth year after kidney transplantation (Banasik et al. 2014). High levels of anti-AT1R and/or anti-ETAR antibodies were associated with morphological and functional allograft injury and graft loss (Banasik et al. 2014). Simultaneous production of anti-HLA antibodies and antibodies directed against nonpolymorphic antigens expressed by the graft was also found, and could contribute to allograft destruction. This implies that a breakdown of self-tolerance occurs during chronic rejection and AMR is a complex interplay between allo- and autoimmune humoral responses (Sicard et al. 2016).

Immunopathologic evidence left by antibodies is C4d deposition in peritubular capillaries. C4d is a fragment of C4b, an activation product of the classic complement pathway. C4d deposition is strongly associated with circulating antibodies to donor HLA class I or class II antigens and is the marker of complement-fixing of circulating antibodies to the endothelium (Böhmig et al. 2002).

Nouël et al. (2014) showed that kidney transplant patients with chronic antibody-mediated rejection display unique B cell phenotype with reduced ratio of activated to memory B cells associated with an impaired immunosuppressive activity of B cells. AMR patients had increased number of memory B cells with potentially upregulated antigen-presenting capacity and decreased percentage and number of transitional B cells with regulatory function. Analysis of B cell compartment could be potentially useful as a biomarker to identify patients at risk of AMR and a guide for therapy in the patients with AMR.

It deserves highlighting that the mechanisms of nephron loss resulting in chronic allograft injury comprise not only immunologic but also non-immunologic factors, such as ischemia–reperfusion injury, calcineurin inhibitor nephrotoxicity, nephron mass, nonadherence to treatment, viral or bacterial infections, proteinuria, hypertension, and hyperlipidemia (Chapman et al. 2005; Pratschke et al. 2008).

Our studies including about 700 patients transplanted between 1990 and 2000 suggest that factors triggering the chronic allograft dysfunction pathomechanisms include renal injury during the perioperative period (which manifested by delayed graft function); older donor age, episodes of acute rejection, and cytomegalovirus (CMV) infections (Boratyńska et al. 2014). In the later phase of chronic allograft injury proteinuria developed along with worsening of arterial hypertension and graft function. These are both symptoms and risk factors of chronic injury progression, and are followed by metabolic disorders typical for chronic kidney disease. This leads to a conclusion that preventing graft injury during the perioperative period as well as protecting the organ against acute rejection and CMV infection may reduce development of chronic allograft injury. Monitoring of proteinuria and implementation of anti-proteinuric therapy, as well as treating hypertension, dyslipidemia, hyperuricemia, and metabolic acidosis might prolong graft function (Nankivell and Kuypers 2011; Renders and Heeman 2012). Immunosuppressive therapy is ineffective in patients with established chronic allograft injury.

A major barrier for improving long-term solid organ allograft result, beside chronic rejection is death of patients with functional graft. Collaborative Transplant Study covering data of more than 157,000 recipients of first kidney transplant from deceased donor transplanted between 1985 and 2000, reported that during 5 years 17% died but within 10 years the percentage rose to 31% of patients (EBPG Expert Group on Renal Transplantation 2002). Three main causes of mortality in transplant recipients: cardiovascular disease, infection and malignancy are associated with side effects of immunosuppressive treatment (Chapman et al. 2013; Kahwaji et al. 2011; Vanrenterghem et al. 2008; Watorek et al. 2011). The induction of immune tolerance could release patients from the need for long-term immunosuppressive treatment.

## Immunologic Tolerance in Solid Organ Transplantation

Transplant immune tolerance is a state of acceptance of allograft without the need of maintenance immunosuppression while the reactivity against all other antigens remains intact, and thus the recipient does not suffer from immunosuppression-related infections and malignancies. The mechanisms underlying tolerance development are still not clear. They can be divided into these leading to “central tolerance” and those leading to “peripheral tolerance” and include ignorance, clonal exhaustion, anergy, deletion and regulation. The regulation is attributed to the regulatory cells, which can downregulate the immune response to antigens of the donor, leading to transplant tolerance.

## Hematopoietic Stem Cell Transplantation for Induction of Allograft Tolerance

The tolerance has been achieved in numerous animal models of transplantation, but in human the induction of long-term tolerance is less successful. Tolerance induction in clinical kidney transplantation from HLA-matched or mismatched live donors may be achieved through the administration of donor antigens in the form of hematopoietic stem cell (HSC) with nonmyeloablative preconditioning approach and gradual tapering of immunosuppression.

This strategy of development of tolerance was used by three centers in the US: Stanford, Northwestern/Louisville and Massachusetts General Hospital, although the protocols differed in many ways (Kawai et al. 2008; Leventhal et al. 2012; Scandling et al. 2011). The basis for allograft tolerance applying HSC was to achieve and maintain donor hematopoietic chimerism for long term, lasting at least several months. However, the loss of chimerism in the peripheral blood did not always predict or lead to graft loss.

The Stanford group recently published results of three cohorts of total 38 patients transplanted between 2000 and 2013 (Scandling et al. 2015). Their conditioning protocol consisted of total lymphoid irradiation (TLI) for 10 days starting on postoperative day 1 and ATG for 5 days. Following the last dose of TLI, CD34^+^-enriched donor peripheral blood stem cells were infused. These cells were collected by apheresis from the donor after treatment with granulocyte colony-stimulating factor. TLI as well as CD34^+^ cells were used in an increasing dose in the second and third cohort. Additionally, CD34^+^ and CD3^+^ T cells (10 and 50 times higher than in prior two cohorts, respectively) were used in the third cohort. The chimerism was achieved only in 7 out of the 22 patients in the second cohort and in 2 of 10 from the third cohort. The chimerism and relative sparing of T regulatory (Treg) versus T effector cells contributed to the development and stability of the tolerance. The successful withdrawal of immunosuppression has been achieved in 16 of 22 patients (72%) with HLA-matched kidney.

Northwestern University trials included 29 recipients; 19 transplanted from HLA mismatched living donors and ten from HLA-matched (Leventhal et al. 2015). In the mismatched group, nonmyeloablative conditioning was used [fludarabine, cyclophosphamide, and total body irradiation (TBI)] combined with HSC and facilitating cells—a mixture of CD8^+^/TCR^−^ to enhance engraftment and reduce the risk of graft-versus-host disease. Twelve of 19 achieved durable chimerism and were off immunosuppression for more than a year, two patients lost their allograft. The HLA-matched group received alemtuzumab and serial infusions of CD34^+^ cells. Microchimerism was achieved for 1 year and 50% of patients were withdrawn from immunosuppression. The persistent chimerism was a better predictor of tolerance than donor-specific hyporesponsiveness tested in vitro.

Massachusetts General Hospital Group conducted a trial including ten recipients transplanted from HLA-haplo matched living donors using thymic irradiation combined with anti-CD2 monoclonal antibodies, rituximab and cyclophosphamide (60 mg/kg bw at day 5 and 4 before kidney transplant) with bone marrow infusion at the day of kidney transplantation (Kawai et al. 2008, 2013). The patients achieved transient mixed chimerism, and in seven patients immunosuppression had been withdrawn although only in four patients for long term. Overall, three patients lost their graft. In the trial most patients developed engraftment syndrome characterized by severe acute renal injury (Farris et al. 2011). Modified version of the protocol with TBI replacing cyclophosphamide, to prevent engraftment syndrome, was used in two other recipients. In the study, deletion of donor reactive T cell clones correlated with the tolerant state.

In all the presented trials, the recipients were strictly monitored for allograft function and immune status, including mixed leukocyte culture, cell mediated cytotoxicity, Tregs, donor reactive T cells (high sequencing of the T cell receptor β chain CDR3 region), quantitation of donor chimerism. Some other registered clinical trials on induction of tolerance in renal, liver and heart transplantation are presented in Table 1.

**Table 1:** Clinical trials the induction of immune tolerance in organ transplantation (ClinicalTrials.gov)

Title of study	Tolerance-inducing strategy	Location and sponsor
Kidney and blood stem cell transplantation that eliminates requirement for immunosuppressive drugs	Kidney HLA-matched from LD; conditioning: TLI + ATG for 2 weeks; The stem cells (CD34) + CD3 removed from donor will be injected at the end of the 2-week treatment; CsA + MMF are stopped if stable chimerism is achieved	Stanford University
Tolerance induction in living donor kidney transplantation with hematopoietic stem cell transplantation	Conditioning: 1 week prior to KT; Rituximab, Fludarabine, Cyclophosphamide, Thymic irradiation; Donor HSCT infused immediately posttransplantation	Samsung Medical Center
Bone marrow transplantation and high dose posttransplant cyclophosphamide for chimerism induction and renal allograft tolerance	Conditioning: ATG, fludarabine, cyclophosphamide 14.5 mg/kg/day, TBI; infusion of bone marrow from donor on day 0; posttransplant: cyclophosphamide 50 mg/kg bw on day 3 and 4; MMF and prednisone	National Institute of Allergy and Infectious Diseases; Collaborator: ITN
Use of belatacept during post depletional repopulation to facilitate tolerance in renal allograft recipients	Kidney from HLA-non-identical living or deceased donor; a single dose of alemtuzumab on the day of transplantation; a single dose of donor bone marrow 7 days after transplantation; belatacept and sirolimus for 1 year	Emory University Atlanta, US; Collaborator: Bristol-Myers Squibb
Autologous hematopoietic stem cell transplantation for allogeneic organ transplant tolerance; (ASCOTT)	3–6 months after liver transplantation; step 1: chemotherapy and cytokine-based treatment for mobilization of HSC, ex vivo purification CD34; step 2: immune ablation (busulphan, cyclophosphamide, ATG) prior to the infusion of HSC	University of Toronto
Induction of donor-specific tolerance in recipients of cardiac allografts by donor stem cell infusion	Bone marrow processed to enrich hematopoietic stem cells and graft facilitating cells. Donor HSC infusion to recipient	University of Louisville; Jewish Hospital and St. Mary’s Healthcare; Hahnemann University Hospital; The Cleveland Clinic
Renal transplantation followed by infusion of T regulatory cells made with belatacept ex vivo (the ONE Study)	Recipients of LD; administration of Treg derived from recipient PBMC, stimulated with kidney donor PBMC in the presence of belatacept; measurement of Treg in the peripheral circulation of kidney recipients. Reduction of immunosuppression	Massachusetts General Hospital Collaborators: Dana-Farber Cancer Institute; University of Regensburg
Safety study of using regulatory T cells induce liver transplantation tolerance (Treg)	1. Group: donor alloantigen-specific Tregs from PB of pretransplant patients, administered (1 × 10^6^ cells/kg) at several intervals; 2. Group: 1–10 year post LD liver transplantation; isolated Tregs from these patients, and expanded with mismatched LD antigens; administration of donor antigen-specific Tregs (1 × 10^6^ cells/kg) at several intervals	Nanjing Medical University; Collaborator: University of Minnesota
Efficacy of low dose, subcutaneous interleukin-2 (IL-2) to expand endogenous regulatory T cells in liver transplant recipients	Liver transplant recipients 2–4 years posttransplantation to receive IL-2 at dose 1.0 MIU/m^2^ body surface area for 4 weeks to expand Tregs	Beth Israel Deaconess Medical Center
The ONE Study: a unified approach to evaluating cellular immunotherapy in solid organ transplantation—M Reg trial	Donor Mreg at dose 2.5–7.5 million cells/kg bw infused into recipients 6–7 days before kidney transplantation from live donor	University of Regensburg
Mesenchymal stem cells (MSC) under basiliximab/low dose RATG to induce renal transplant	Infusion of syngenic ex vivo expanded MSCs (2 × 10^6^/kg bw) at the time of kidney transplantation from LD under basiliximab/low dose ATG induction therapy and maintenance CsA, MMF and prednisone	Mario Negri Institute for Pharmacological Research, Italy
Third-party bone marrow-derived mesenchymal stromal cells to induce tolerance in liver transplant recipients	In liver transplant: a single intravenous infusion (1–2 millions of MSCs/kg bw) of ex vivo expanded third-party MSC (from healthy donors)	Monia Lorini, Mario Negri Institute for Pharmacological Research

*TBI* total body irradiation, *TLI* total lymphoid irradiation, *LD* living donor, *KT* kidney transplantation, *HSCT* hematopoietic stem cell transplantation, *Mreg* regulatory macrophages, *PB* peripheral blood, *MSC* mesenchymal stromal cells, *ATG* anti-thymocyte globulin, *CsA* cyclosporine A, *MMF* mycophenolate mofetil, *ITN* Immune Tolerance Network

## Immunoregulatory Cells in Tolerance Induction

The alternative method of inducing of transplantation tolerance may be the administration of regulatory cells (Geissler 2012; Scalea et al. 2016). Several experimental studies strongly support that tolerance is mediated by immunoregulatory cells. Recently, there has been a great interest in the regulatory cell-based therapy because of the ONE Study (“A Unified Approach to Evaluating Cellular Immunotherapy in Solid Organ Transplantation”) funded by the European Commission’s Seventh Framework. There, seven different regulatory cell populations have been tested as possible routes to tolerance induction (Geissler 2012). The allograft recipients have been treated with a concentrated dose of well-defined regulatory immune cells near the time of transplantation, which is supposed to trigger a self-sustaining immune regulation. The multicenter trial (involving clinical centers in France, Germany, Italy, UK, and US) assesses naturally occurring regulatory T cells (nTreg), alloantigen-driven Treg, mesenchymal stem cells, regulatory macrophages, dendritic regulatory cells, and myeloid derived regulatory cells (Elias et al. 2015). Immunosuppression protocol has been the same at all the trial sites and includes tacrolimus, mycophenolate mofetil and prednisone allowing comparison of outcomes.

The primary interest was focused on the Tregs, whose suppressive role in vivo is well documented and widely discussed for two decades (Bushell et al. 1995). There are many subsets Tregs; thymus-derived naturally occurring Tregs are required for self-tolerance. The common feature of Tregs is the expression of transcription factor forkhead boxP3 (FoxP3). CD4^+^CD25^+^ FoxP3 Tregs promote and maintain allograft tolerance in animal models (Juvet et al. 2014). They induce regulatory properties in alloreactive T cells and may prevent chronic allograft injury. Tregs utilize multiple mechanisms to inhibit effector T cells, which include modulation of antigen-presenting cells (APC) function; metabolic disruption (IL-2 deprivation, adenosine secretion); direct cytotoxicity toward effector T cells; direct cell-to-cell interaction; and secretion of inhibitory cytokines, such as IL-10, IL-35, and TGF-β. Tregs mediated allograft response in secondary lymphoid organs and in the graft itself (Rothstein and Camirand 2015). Tolerated grafts are infiltrated by recipient lymphocytes that restrain local immune responses. Moreover, Tregs within the allograft may regulate tissue homeostasis and metabolism and may contribute to tissue repair.

Investigators from Massachusetts General Hospital found that tolerant phenotype is associated with the persistence an increased proportion of CD4^+^CD25^+^CD127^−^FoxP3^+^ Treg during early posttransplant period of induction tolerance (Andreola et al. 2011). Braza et al. (2015) reported that in tolerant recipients the Tregs exhibited increased FoxP3 demethylation of the Treg-specific demethylated region and increased suppressive properties in vitro compared with healthy volunteers, patients with stable allograft function receiving immunosuppression and those with chronic rejection.

The low quantity of Tregs in peripheral blood necessitates ex vivo expansion prior to clinical use. Several methods for the expansion of CD4^+^CD25^+^CD127^low^ Tregs from peripheral blood have been developed. Most protocols use anti-CD3/CD28 antibodies coated to beads, artificial APC expressing high affinity Fc, and recombinant human IL-2. Expanded ex vivo Tregs and returned to the patients retained Foxp3 expression that could be detected for at least 30 days.

The first clinical study with Treg in clinical renal transplantation was designed within the ONE Study and entitled: “Infusion of T-Regulatory Cells in Kidney Transplant Recipients”. In this trial, Tregs were derived from recipient peripheral blood mononuclear cells (PBMC) and stimulated with kidney donor PBMC. Under these conditions, the expansion was achieved by costimulatory blockade. The donor-alloantigen-reactive Tregs have been given back to the recipient 7–10 days after transplantation (Juvet et al. 2014; Rothstein and Camirand 2015). The study examines the safety and feasibility of administering Tregs in renal transplant recipients from living donor. Moreover, it is to define whether administration of the Treg cell product allows for tapering of immunosuppressive drugs within 60 weeks after transplantation. During the period of study the presence, potency, and specificity of Treg in the peripheral circulation of each kidney transplant recipient has been assayed. Selected, registered clinical trials on Tregs are listed in Table 1.

Another interesting option for cell-based tolerance induction in human recipients is regulatory macrophages (Mregs) (Scalea et al. 2016). Human Mregs inhibit T cell proliferation via interferon (IFN)-γ-induced indoleamine 2,3-dioxygenase activity and delete activated T cells. Riquelme et al. (2013) reported that one dose of donor-derived Mregs given 8 days before cardiac transplantation in mice prolonged allograft survival in immunocompetent recipients. Clinical trial TAIC-II (transplant acceptance-inducing cells) assessed the safety and efficacy of administering Mreg in five recipients of living donor renal transplant. Mregs were obtained by culturing donor PBMCs in macrophage colony-stimulating factor and stimulation with IFN-γ followed by coculture with recipient PBMCs. The patients received induction therapy with ATG in addition to steroid and tacrolimus. Mreg were infused at dose 1.4–5.9  ×  10^8^ cells. A total three out of five patients experienced allograft rejection during weaning or after withdrawal immunosuppression (Hutchinson et al. 2008). Other clinical trials with different regulatory cells in solid organ transplantation are presented in Table 1.

Woodward et al. (2016) presented recently an attractive concept of the tolerance induction without the use of chronic immunosuppression by the manipulation of the graft, rather than the recipient. In this approach, grafts are engineered with immunomodulatory molecules. This technology involves changing the ratio of T effector versus CD4^+^CD25^+^FoxP3^+^ T regulatory cells within the graft microenvironment. As a result, localized tolerance is expected.

## Biomarkers of Tolerance

A major factor limiting the broader clinical application of strategies to induce tolerance is the lack of markers to predict and identify the tolerance in patients. The reliable markers would increase the safety of tolerance studies and also may aid in the design of tolerance-inducing immunosuppressive protocol. Two multicenter studies from Europe and the US (Indices of Tolerance/RISET and Immune Tolerance Network) including 36 patients with operational tolerance (defined by stable graft function despite cessation of immunosuppression for more than 1 year, usually because of nonadherence) found that these patients have a unique pattern of cells and genes expressed in their blood compared to other transplant patients (Newell et al. 2010; Sagoo et al. 2010). Tolerant renal transplant patients showed expression of multiple B cell differentiation genes in the peripheral blood and a set of three of these genes (TCL1A, CD20, CD79b) distinguished tolerant from nontolerant recipients. The B cell signature was associated with upregulation of CD20 mRNA in urine sediment cells. In addition, the increase of total number of B cells and defects in B cell maturation, as result of the increase of transitional B cells (CD19^+^CD38^+^CD24^+^IgD^+^), naïve B cells (CD19^+^CD27^−^IgD^+^), and memory B cells were found. Tolerant recipients exhibited lower number of CD4^+^ cells, decreased production of IFN-γ in ELISPOT and high ratio of FoxP3 expression to α-1,2-mannosidase gene expression in peripheral blood cells. Biomarkers associated with tolerance following liver transplantation are different. They were found to be associated to NK cells, γ/δ T cells in the blood, and genes related to iron homeostasis in the graft (Newell and Turka 2015). The tolerance biomarkers will require validation on prospective, larger studies of transplant recipients undergoing minimization or withdrawal of immunosuppression.

The scientists participating in main clinical tolerance trials in the report titled “Tolerance: One Transplant for Life” presented consensus and recommendation that make tolerance induction protocols a standard of care for transplant recipients over the next decade (Kawai et al. 2014). The major recommendations include to establish standards of registering patients and reporting the results of tolerance trials (functional organ status, histologic findings); to establish a standard panel of biomarkers of tolerance; to standardize protocols for sample collection and storage; and to include children 12 years and older into tolerance induction study.

Major impediment to progress is high costs of tolerance trials. Costs for tolerance studies are up to 300,000 $ per patient (it is six times higher than in drug safety and efficacy trials). These account conditioning, administering HSCT or regulatory cells, monitoring of patients, use of nonstandard procedures and longer term of follow-up (Kawai et al. 2014).

Many barriers exist for introduction protocols inducing tolerance to transplant clinic. There is need of new protocols, new trials that involve more centers and larger number of patients and extend protocols of induction tolerance to other organs and to organs from deceased donors.

## Xenotransplantation

The dream of animal organs and tissue as a transplant material in clinical use is not young. We had such dramatically clinical situations in Poland also, with pig heart transplanted to human recipient by Religa in the lack of human donor in the late 80s. All those early efforts constructed the wide field of scientific work in this domain. In 2016 ISODP Congress in Seoul, we had heard the perspective of 2030 to see the first animal organ transplanted to human recipient. Progress done in genetically modified pigs shows such perspective in a real light but does not answer many other questions arose (Cooper et al. 2016; Olver 2016). When most problems concerned about immunotolerance and immunosuppression in xenotransplantation seems to be possible to be solved quite soon, than thrombogenicity and problems of unknown infectious dangers are still before us (Cooper et al. 2016).

## Regenerative Medicine

Both xenotransplantation and regenerative medicine promise to free clinical transplantation from all dilemmas connected with human donation of organs and cells. Last decade give us hope for such solutions. The idea of using own recipient cells cultured on the natural or 3D printed matrix scaffold is intensively introduced in basic science and some models (urine bladder) are in clinical trials in more than 10 years, with very promising results (Jung et al. 2016; Zhang et al. 2016).

## Transplant Staff Burning-Out Syndrome

There are not a big number of specific papers describing situation of transplant staff. The transplant centers in Europe and North America are using more and more foreign doctors and nursing staff in transplantation and other specialities. In clinical transplantation, the unlimited (in practice) work time and unpredictability makes difficult to recruit the new candidates to this field. Polish solution with a mandatory specialization in clinical transplantology (4–5 years of additional training) makes this problem even more dangerous. Even without that problem, a dramatical decrease in the nursing staff in all Polish hospitals needs immediate and wise decisions now (Le Gall 2011).

Other challenges in clinical transplantation as organization and financing are not in the scope of this paper.
